# Epinephrine Injection Associated Scrotal Skin Necrosis

**DOI:** 10.1155/2015/187831

**Published:** 2015-06-22

**Authors:** Murat Gul, Mehmet Kaynar, Tamer Sekmenli, Ilhan Ciftci, Serdar Goktas

**Affiliations:** ^1^Department of Urology, Van Training and Research Hospital, 65100 Van, Turkey; ^2^Department of Urology, Selcuk University School of Medicine, Turkey; ^3^Department of Pediatric Surgery, Selcuk University School of Medicine, Turkey

## Abstract

Male circumcision is among the most frequent surgical interventions throughout history. Although considered as a minor intervention, it may have complications ranging from insignificant to catastrophic. These complications can be attributed to the surgical procedure and anesthesia. In this report we present two cases of scrotal skin necrosis after lidocaine with epinephrine injection using subcutaneous ring block technique prior to circumcision.

## 1. Introduction

Male circumcision, excising of the foreskin of penis, for traditional, religious, medical, or personal reasons is the most frequently applied surgical procedure in history. Although it can be considered as a minor surgery, it may lead to complications ranging from insignificant to catastrophic [1]. These complications can be attributed to both surgical procedure and anesthesia.

Today, circumcision is performed using several local anesthetic techniques such as caudal epidural block [2], dorsal penile nerve block (DPNB) [3], subcutaneous ring block (SRB) [4], and topical anesthesia applications [5]. We present two cases of scrotal skin necrosis after lidocaine with epinephrine injection using SRB technique prior to circumcision.

The guardians of both cases reviewed the case report and gave their written consent to the corresponding author for publication of the present report.

## 2. Case 1

**Figure 1:**
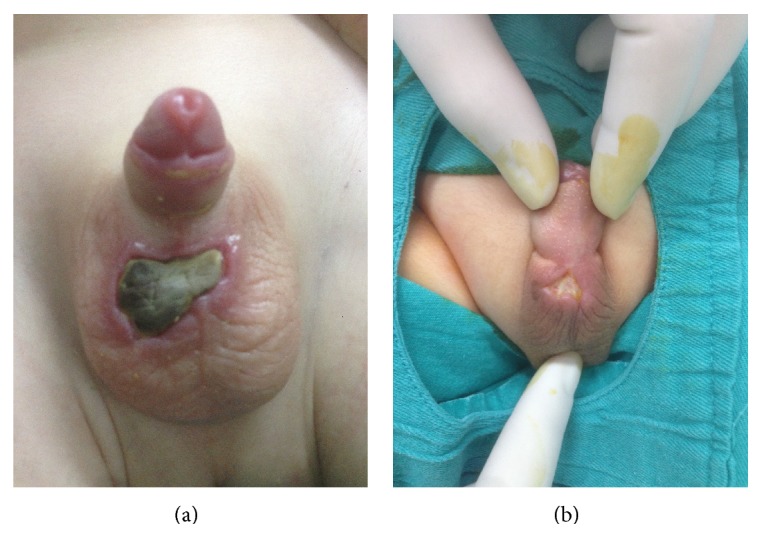
(a) Skin necrosis with a crusty wound just below the penoscrotal junction following lidocaine injection in case 1. (b) Skin necrosis with a fallen shell wound just below the penoscrotal junction following lidocaine injection in case 2.

**Figure 2:**
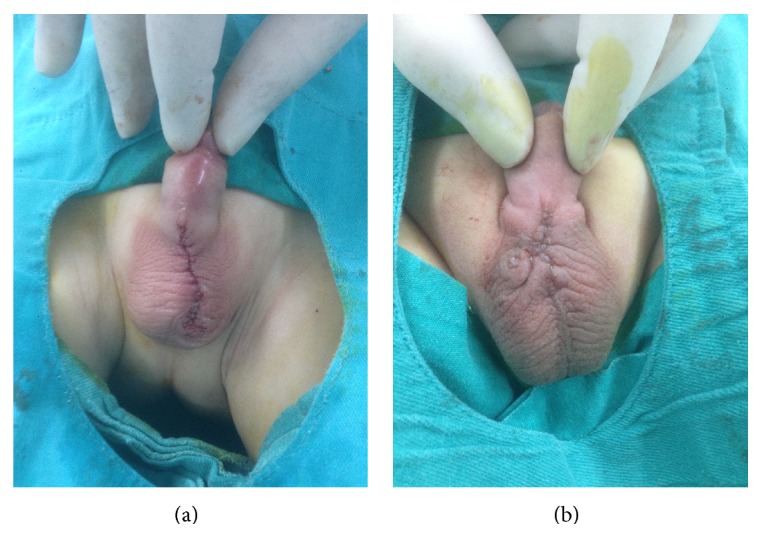
(a) Appearances of case 1 and (b) case 2 following second surgery.

A 2-year-old toddler was referred to our clinic with micturition complaints. His physical examination revealed phimosis and immediate circumcision was decided. Blood test was normal (PT, PTT, and INR). Prior to circumcision, local anesthesia was performed with lidocaine 1% containing epinephrine 1 : 200,000 subcutaneously using subcutaneous ring block anesthesia technique by a 27-gauge conventional Tuberculin Syringe. On the 5th postoperative day the patient was brought into the clinic with a crusty wound just below the penoscrotal junction. Conservative wound management was attempted using I.V. antibiotics and anti-inflammatory treatment. Sterile culture from the site of necrosis was obtained (Figure 1(a)). However, surgical intervention was decided when the site of necrosis enlarged one week later. Under laryngeal mask anesthesia necrotic lesion and tissue were excised and incision closed vertically (Figure 2(a)). During the control on the 6th postoperative day, it was seen that the patient's wound healed completely without any complication.

## 3. Case 2

A one-year-old toddler was admitted to our clinic with micturition complaints as in case 1 (Figure 1(b)). His circumcision was made using the same local anesthetic drug and technique. After 4 days patient was brought in with a fallen shell wound just below the penoscrotal junction. In this case, no conservative management was attempted and the second surgery was performed immediately. His wound also healed completely without any complication (Figure 2(b)).

## 4. Discussion

Circumcision indisputably is worldwide the most frequently performed minor male surgery [6]. Like any other surgical intervention, circumcision may have complications related to either surgery or anesthesia. These are bleeding, infection, skin bridge, urinary retention, meatitis, chordee, lymphedema, fistulas, and necrosis. Among these, the most common ones are easily handled bleeding and infections. But sometimes serious complications need surgical or reconstructive interventions [7]. In the relevant departments, within the last five years around 500 circumcisions were performed without major complications.

Necrosis following circumcision is a rare complication of glans penis due to infection, local anesthesia solutions with epinephrine, tight dressing, and reckless cauter use [8]. In the present cases skin necrosis developed on 5th and 4th postcircumcision day, respectively. Local anesthesia containing epinephrine is considered to be the underlying reason. There are some contradictory reports about the usage of epinephrine combined with or without lidocaine using SRB technique for circumcision. Epinephrine use for penile block is considered to be contraindicative. Actually a vast number of studies reported penile block without epinephrine [9]. On the other hand, there are some large series that have reported the safe use of epinephrine [10, 11].

More recent studies showed that penis has a more complicated vascular system than earlier considered and less terminal vessels. Hence, epinephrine combined with lidocaine use has been considered as a safe SRB technique prior to circumcision [9]. Therefore, today epinephrine administration with local anesthetics has regained wide currency. Besides, due to the ease of use and prolonged postoperative pain control local anesthesia combination was preferred in the present cases. Previously, no scrotal skin necroses were experienced.

Circumcision might be performed with different local analgesic applications than the one described previously. These are topically applied lidocaine-prilocaine cream, dorsal penile nerve bloc (DPNB), and SRB. Yet, there are some reports that topical use of lidocaine-prilocaine and DPNB with prilocaine may lead to methemoglobinemia [5, 12]. Lidocaine usage on skin may also result in some complications including skin redness, rash, dry skin, blister, contact, and exfoliative dermatitis [13].

Normally SRB is defined as a subcutaneous circumferential ring injection of local anesthetic at the mid shaft of penis and SRB with lidocaine was shown to be the most effective anesthetic technique through all stages of the circumcision [14]. Therefore in our clinics this technique is preferred and applied as in these two cases. To our best knowledge, these are the first scrotal skin necrosis cases developing after local anesthetics' injection for circumcision analgesia. Therefore in the first case a conservative approach was attempted with I.V. antibiotics and anti-inflammatory treatment. However, the site of necrosis enlarged and limited itself but did not show any sign of healing. Eventually necrotic site was removed and complete recovery achieved after second surgery. Our experience with the first case made us avoid conservative treatment attempt and move on with the second surgical intervention.

## 5. Conclusion

Although circumcision using epinephrine is considered as a safe and painless procedure, epinephrine injection associated scrotal skin necrosis of the injection site should not be disregarded. Hence, general anesthesia with caudal blocks for postoperative pain relief can be considered in appropriate cases.
